# Effects of upadacitinib on enthesitis in patients with psoriatic arthritis: a *post hoc* analysis of SELECT-PsA 1 and 2 trials

**DOI:** 10.1093/rheumatology/keae057

**Published:** 2024-02-08

**Authors:** Fabrizio Cantini, Antonio Marchesoni, Lucia Novelli, Giuliana Gualberti, Francesca Marando, Erin L McDearmon-Blondell, Tianming Gao, Dennis McGonagle, Carlo Salvarani

**Affiliations:** Department of Rheumatology, Azienda USL Toscana Centro, Hospital of Prato, Prato, Italy; Department of Rheumatology, Humanitas San Pio X, Milan, Italy; Medical Department, AbbVie srl, Rome, Italy; Medical Department, AbbVie srl, Rome, Italy; Medical Department, AbbVie srl, Rome, Italy; AbbVie Inc, North Chicago, IL; AbbVie Inc, North Chicago, IL; Leeds Institute of Rheumatic and Musculoskeletal Medicine, University of Leeds, Leeds, UK; Leeds Teaching Hospitals NHS Trust, NIHR Leeds Biomedical Research Centre, Leeds, UK; Unità Operativa di Reumatologia, Azienda USL-IRCCS di Reggio Emilia, Reggio Emilia, Italy; Università di Modena e Reggio Emilia, Reggio Emilia, Italy

**Keywords:** psoriatic arthritis, enthesitis, entheses, upadacitinib, Janus kinase inhibitor, pain

## Abstract

**Objectives:**

To characterize the effect of upadacitinib 15 mg once daily (UPA15) on enthesitis in patients with PsA from the SELECT-PsA Phase 3 trials.

**Methods:**

Patients with an inadequate response/intolerance to one or more non-biologic DMARD (SELECT-PsA 1) or one or more biologic DMARD (SELECT-PsA 2) received UPA15, adalimumab 40 mg every other week or placebo (weeks 0–24) switched to UPA15 (week 24 onward). The Leeds Enthesitis Index (LEI) and Spondyloarthritis Research Consortium of Canada (SPARCC) index were used to assess improvement in enthesitis, enthesitis resolution, maintenance of enthesitis resolution and protection from enthesitis development through week 56.

**Results:**

Data from 639 patients receiving UPA15 and 635 patients receiving placebo (including 317 patients who switched from placebo to UPA15) were analysed. UPA15 led to higher rates of enthesitis resolution *vs* placebo at week 24 (LEI: 59.8% *vs* 38.0%; SPARCC index: 50.6% *vs* 31.5%, respectively) and greater improvements in the LEI (–1.7 *vs* –1.0) and SPARCC index (–3.4 *vs* –1.9); improvements were maintained through week 56. Improvements were observed after 12 weeks of UPA15 treatment. Over 90% of patients without enthesitis (LEI = 0) at baseline receiving UPA15 were enthesitis-free at week 56, and UPA15 prevented recurrence of enthesitis at week 56 in >80% of patients with enthesitis at baseline who achieved resolution (LEI = 0) at week 24.

**Conclusions:**

UPA15 is associated with a comprehensive improvement in enthesitis, with improvements observed after 12 weeks of treatment. Additionally, treatment with UPA15 was associated with maintaining an enthesitis-free state after resolution and protection against new-onset enthesitis.

**Trial registration:**

ClinicalTrials.gov identifiers: NCT03104400 (SELECT-PsA 1) and NCT03104374 (SELECT-PsA 2).

Rheumatology key messages

- Upadacitinib 15 mg once daily provided a comprehensive improvement in enthesitis across individual entheseal sites.
- Improvements in enthesitis were observed after 12 weeks of upadacitinib treatment.
- Upadacitinib was associated with a maintained enthesitis-free state after resolution, and protection against new-onset enthesitis.

## Introduction

Enthesitis is the inflammation of entheses (sites of attachment between a tendon, ligament, fascia or capsule, and bone). It is thought to be among the primary developmental events of SpA and is a hallmark manifestation of PsA [1], affecting ∼30% of patients [2]. Compared with patients with PsA without enthesitis, PsA with symptomatic enthesitis is associated with greater disease activity and patient-reported fatigue and pain, as well as worse functional status, work impairment, lower quality of life and reduced likelihood of achieving minimal disease activity [1, 3].

The 2019 treatment guidelines from the EULAR and the Group for Research and Assessment of Psoriasis and Psoriatic Arthritis (GRAPPA) recommend NSAIDs and local glucocorticoids as first-line treatment for enthesitis [4, 5]. In patients with an inadequate response, intolerance or contraindication to NSAIDs, biologic DMARDs (bDMARDs) targeting TNF, IL-17, IL-23 or IL-12/23, or targeted synthetic DMARDs targeting Janus kinases (JAKs) or phosphodiesterase-4, should be considered [4, 5]. Conventional synthetic DMARDs (csDMARDs) are not usually recommended for resolution of enthesitis [4], except MTX, which is conditionally recommended by the GRAPPA guidelines [5]. Updated EULAR guidelines are expected in 2024.

The clinical use of agents such as JAK inhibitors has substantially improved understanding of the pathways underlying development of enthesitis [6]. Upadacitinib, an oral, selective and reversible JAK inhibitor, is approved [15 mg once daily (QD)] for treatment of adults with active PsA and inadequate response or intolerance to one or more DMARD (Europe and other countries) or one or more TNF inhibitor (USA) [7, 8]. In the SELECT-PsA 1 and 2 studies, resolution of enthesitis [defined as Leeds Enthesitis Index (LEI) = 0] was achieved by a significantly greater proportion of patients receiving upadacitinib 15 mg QD compared with placebo at week 24 [9, 10]. Greater proportions of patients with resolution of enthesitis at week 24 were also observed with upadacitinib *vs* placebo using the Spondyloarthritis Research Consortium of Canada (SPARCC) enthesitis index = 0 [9, 10]. In the long-term open-label extensions of SELECT-PsA 1 and 2, the benefits of upadacitinib on enthesitis were maintained for up to 2 years [11, 12]. The objective of this *post hoc* analysis was to characterize further the effect of upadacitinib 15 mg QD, the approved upadacitinib dose for the treatment of PsA [7, 8], on enthesitis in these studies.

## Methods

### Study design

The study designs of the randomized, Phase 3, placebo-controlled SELECT-PsA 1 and 2 studies have been described elsewhere [9, 10]. Briefly, in SELECT-PsA 1, patients with active PsA and an inadequate response or intolerance to one or more non-bDMARD were randomized to placebo, upadacitinib 15 mg QD, upadacitinib 30 mg QD or adalimumab 40 mg every other week [9]. In SELECT-PsA 2, patients with active PsA and an inadequate response or intolerance to one or more bDMARD were randomized to placebo, upadacitinib 15 mg QD or upadacitinib 30 mg QD [10]. In both studies, patients initially randomized to placebo switched to upadacitinib 15 mg QD or upadacitinib 30 mg QD at week 24, with blinding maintained until week 56. This *post hoc* analysis was conducted on data collected from SELECT PsA-1 and 2 trials (NCT03104400 and NCT03104374) which were conducted according to the International Conference on Harmonisation guidelines and the Declaration of Helsinki. The trial protocols were approved by independent ethics committees and institutional review boards, and the *post hoc* analysis did not require any further ethical approval. Written informed consent was provided by patients ahead of study screening.

### Analysis population

For this *post hoc* analysis, data from both studies were pooled by treatment sequence, with outcomes assessed in patients receiving: (i) placebo from baseline to week 24 (placebo group), which included data up to and including week 24 from patients who subsequently received either upadacitinib 15 mg QD or upadacitinib 30 mg QD from week 24; (ii) placebo from baseline to week 24, followed by upadacitinib 15 mg QD from weeks 24–56 [i.e. data from the subset of patients in group (i) following the switch to upadacitinib 15 mg QD at week 24; placebo to upadacitinib 15 mg group]; (iii) continuous upadacitinib 15 mg QD from baseline to week 56 (upadacitinib 15 mg group); or (iv) continuous adalimumab 40 mg every other week. Data from patients who switched from placebo to upadacitinib 30 mg at week 24 were included only in the placebo analysis before the switch because this dose is not approved for the treatment of PsA.

### Assessments

Resolution of enthesitis, defined as the proportion of patients achieving a score of zero on the LEI or SPARCC index, was assessed at weeks 12, 16, 24, 36 and 56 among patients with enthesitis at baseline (defined as LEI or SPARCC index >0) in each treatment group (including adalimumab 40 mg every other week). In patients achieving enthesitis resolution at week 24, maintenance of resolution was assessed at weeks 36 and 56. In patients not achieving enthesitis resolution at week 24, the locations of residual enthesitis were further evaluated. Changes from baseline in LEI and SPARCC index were also assessed in patients with baseline LEI or SPARCC index >0. In addition, protection from enthesitis development (LEI = 0) over time was assessed in patients without enthesitis (LEI = 0) at baseline.

Patients’ assessment of pain was assessed over time in patients with enthesitis (SPARCC index >0) at baseline in each treatment group, including adalimumab 40 mg every other week. This was also evaluated in the subgroups who achieved or did not achieve resolution at week 24 (SPARCC index = 0 or >0, respectively). The SPARCC index was used for this analysis, as it requires the evaluation of more entheseal sites than the LEI (18 *vs* 6, respectively), and is therefore considered to be a more sensitive measure of enthesitis [13].

### Statistical analyses

This was a *post hoc* analysis. Non-responder imputation (NRI) with additional rescue handling was applied to binary endpoints, with patients who received rescue therapy at week 16 imputed as non-responders in all analyses after week 16. As observed data are also shown for binary endpoints, with nominal *P*-values calculated using the Cochran–Mantel–Haenszel test adjusting for study and current non-bDMARD use. Mixed model for repeated measures was used for continuous endpoints; the models included fixed factors for study, treatment, visit, the interaction of treatment and visit, and current non-bDMARD use, with baseline measurement as a fixed covariate. Pearson correlation was used to assess the correlation between enthesitis and pain.

## Results

### Patients

Data from 639 patients who received upadacitinib 15 mg and 635 patients receiving placebo (including 317 patients who switched from placebo to upadacitinib 15 mg) were included in this analysis. For the pain analysis, 322 patients with SPARCC index >0 were included for the adalimumab 40 mg every other week group. At baseline, similar proportions of patients in each treatment group had enthesitis, ranging from 59.6% to 63.1% for patients with LEI >0 and 77.0–78.2% for patients with SPARCC index >0 (Supplementary Table S1, available at *Rheumatology* online).

Baseline characteristics were generally well balanced in the overall population, across treatment groups, and in patients with or without enthesitis at baseline (regardless of assessment with LEI or SPARCC; Supplementary Table S2, available at *Rheumatology* online). However, numerically more patients with enthesitis were female, and had a higher BMI and tender joint count than patients without enthesitis.

### Resolution of enthesitis through week 56

**Figure 1.**
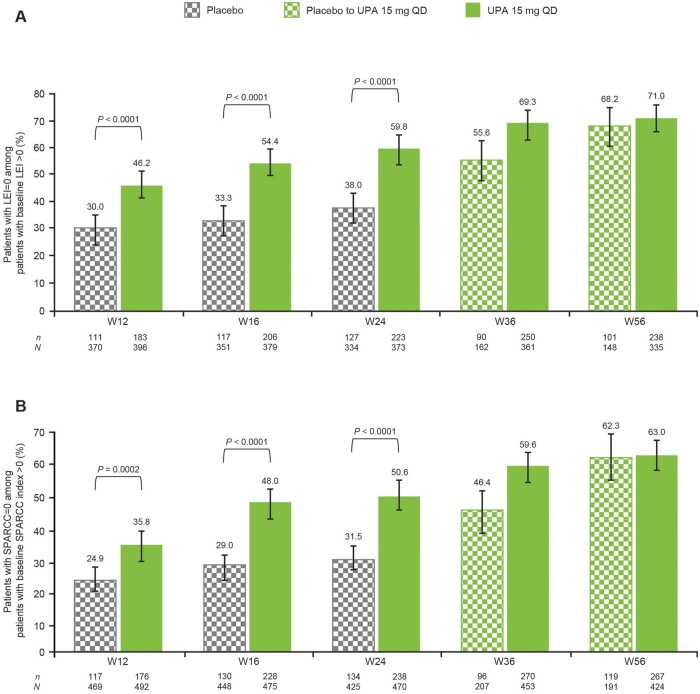
Resolution of enthesitis over time in patients with enthesitis at baseline (**A**) LEI and (**B**) SPARCC index (as observed). Error bars represent 95% CIs. All *P*-values are nominal. LEI: Leeds Enthesitis Index; QD: once daily; SPARCC: Spondyloarthritis Research Consortium of Canada; UPA: upadacitinib; W: week

A higher proportion of patients with enthesitis at baseline in the upadacitinib 15 mg group than in the placebo group achieved resolution of enthesitis at weeks 12 (LEI: 46.2% *vs* 30.0%; SPARCC index: 35.8% *vs* 24.9%, respectively), 16 (LEI: 54.4% *vs* 33.3%; SPARCC index: 48.0% *vs* 29.0%, respectively) and 24 [LEI: 59.8% *vs* 38.0%; SPARCC index: 50.6% *vs* 31.5%, respectively; nominal *P* *<* 0.0001 at each time point for each measure, except for the SPARCC index at week 12 (*P* *=* 0.0002); Fig. 1A and B]. Following the switch from placebo to upadacitinib 15 mg, the proportion of patients with no enthesitis (LEI = 0) increased from 38.0% at week 24 (pre-switch) to 55.6% at week 36 (after 12 weeks of upadacitinib treatment). At week 56 (after 32 weeks of upadacitinib treatment), the proportion of patients without enthesitis in this group (68.2%) was similar to the proportion in the upadacitinib 15 mg group who received 56 weeks of continuous upadacitinib (71.0%; Fig.1A). Similar patterns were seen for SPARCC index = 0 (Fig. 1B). In patients receiving adalimumab 40 mg every other week, 39.4%, 43.2% and 51.6% of 322 patients with enthesitis at baseline achieved resolution of enthesitis at weeks 12, 24 and 56, respectively (Supplementary Fig. S1, available at *Rheumatology* online).

### Change from baseline in LEI and SPARCC scores through week 56

**Figure 2.**
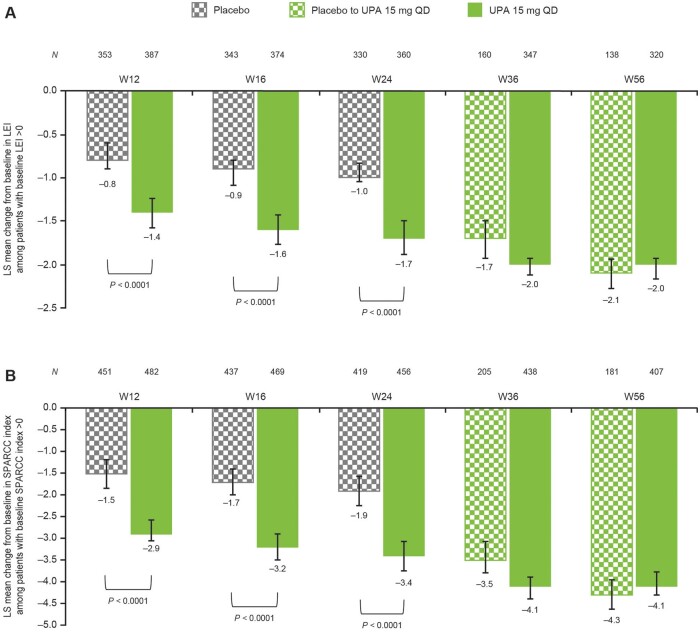
Change from baseline in (**A**) LEI and (**B**) SPARCC index in patients with baseline enthesitis (MMRM). Error bars represent 95% CIs. All *P*-values are nominal. LEI: Leeds Enthesitis Index; LS: least squares; MMRM: mixed model for repeated measures; QD: once daily; SPARCC: Spondyloarthritis Research Consortium of Canada; UPA: upadacitinib; W: week

There was an improvement from baseline in LEI and SPARCC index in the upadacitinib 15 mg group compared with the placebo group at weeks 12, 16 and 24 (nominal *P* *<* 0.0001 at each time point; Fig. 2). This improvement was maintained through week 56, with least-squares mean changes from baseline in LEI score at week 36 of –2.0 and –1.7 in the upadacitinib 15 mg and placebo to upadacitinib 15 mg groups, respectively, and at week 56 of –2.0 and –2.1, respectively (Fig. 2A). In addition, by week 56, patients in the placebo to upadacitinib 15 mg group demonstrated generally similar changes from baseline in LEI as those in the upadacitinib 15 mg group (Fig. 2A). Similar results were seen in terms of SPARCC index (Fig. 2B).

### Maintenance of an enthesitis-free state after resolution

**Figure 3.**
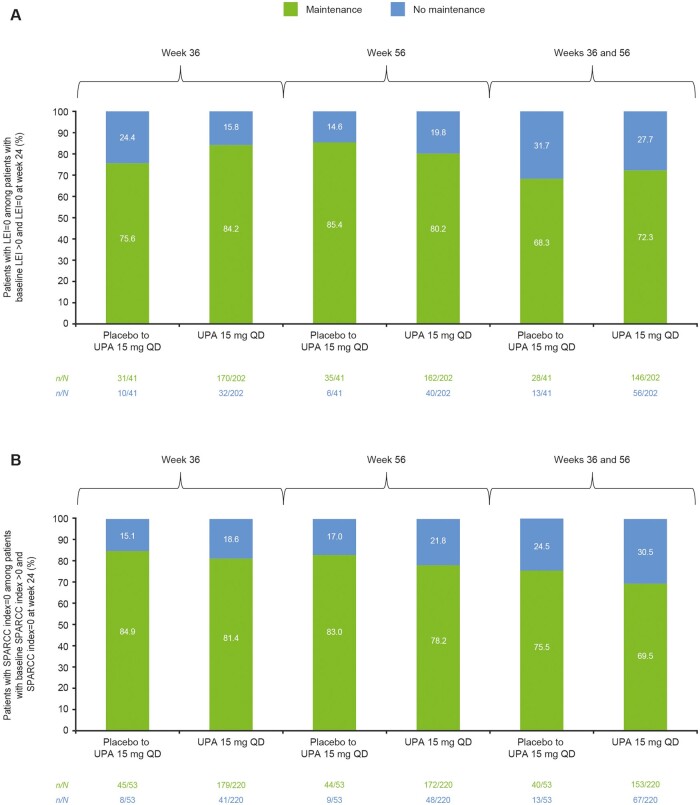
Maintenance of enthesitis resolution in patients with resolution at week 24 and baseline enthesitis, (**A**) LEI and (**B**) SPARCC index (NRI). LEI: Leeds Enthesitis Index; NRI: non-responder imputation; QD: once daily; SPARCC: Spondyloarthritis Research Consortium of Canada; UPA: upadacitinib

Among patients who achieved resolution of enthesitis (LEI = 0) at week 24, resolution was maintained at week 56 in 85.4% of patients in the placebo to upadacitinib 15 mg group and 80.2% in the upadacitinib 15 mg group (NRI data; Fig. 3A). Similar results were obtained using SPARCC index = 0 for enthesitis resolution (83.0% and 78.2%, respectively; Fig. 3B).

### Location of residual enthesitis sites at week 24

**Figure 4.**
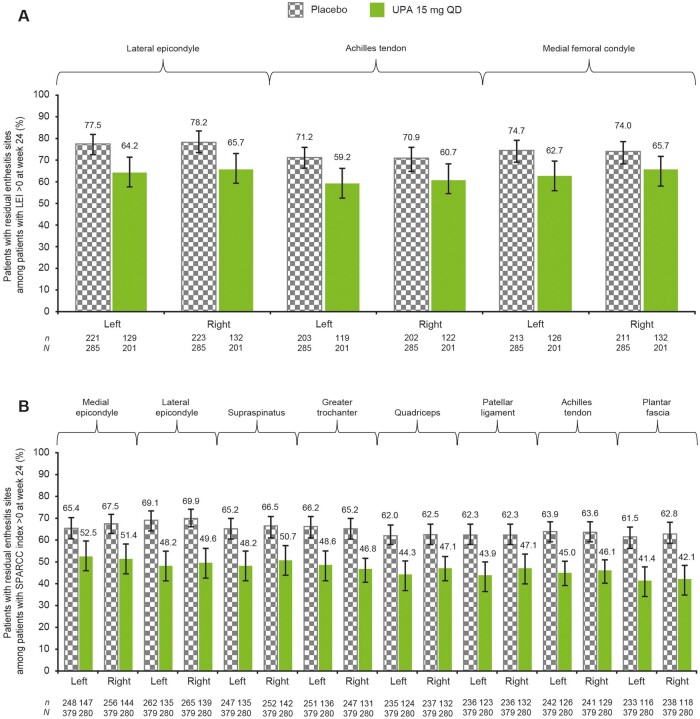
Residual enthesitis sites at week 24 in patients without resolution of enthesitis, (**A**) LEI and (**B**) SPARCC index (NRI). Error bars represent 95% CIs. LEI: Leeds Enthesitis Index; NRI: non-responder imputation; QD: once daily; SPARCC: Spondyloarthritis Research Consortium of Canada; UPA: upadacitinib

Among patients with residual enthesitis at week 24, numerically lower proportions of patients experienced enthesitis at each site measured by the LEI (Fig. 4A) and the SPARCC index (Fig. 4B) in the upadacitinib 15 mg group compared with the placebo group. No specific refractory sites were identified in either group.

### Protection from enthesitis development over time

**Figure 5.**
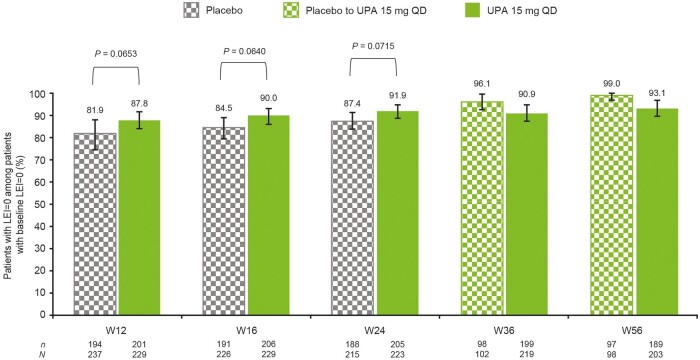
Protection from enthesitis development over time in patients without enthesitis at baseline (as observed). Error bars represent 95% CIs. All *P*-values are nominal. LEI: Leeds Enthesitis Index; QD: once daily; UPA: upadacitinib; W: week

A numerically higher proportion of patients without enthesitis (LEI = 0) at baseline in the upadacitinib 15 mg group remained enthesitis-free at week 24 compared with the placebo group [91.9% and 87.4%, respectively (as observed data); Fig. 5]. At weeks 36 and 56, protection from enthesitis development was observed in >90% of patients in both the upadacitinib 15 mg and placebo to upadacitinib 15 mg groups, with similar proportions of patients enthesitis-free at each time point in both groups (90.9% and 93.1%, respectively, at weeks 36 and 56 with upadacitinib 15 mg, and 96.1% and 99.0%, respectively, for placebo to upadacitinib 15 mg). Similar patterns were seen using NRI (data not shown).

### Correlation between pain and enthesitis

**Figure 6.**
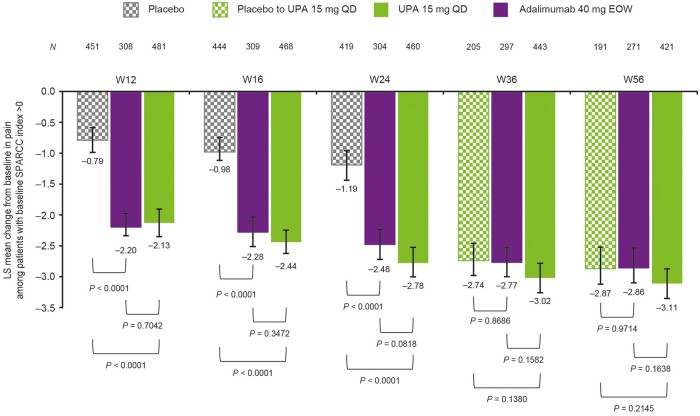
Patient’s assessment of pain in patients with enthesitis at baseline (MMRM). Error bars represent 95% CIs. All *P*-values are nominal. EOW: every other week; LS: least squares; MMRM: mixed model for repeated measures; QD: once daily; SPARCC: Spondyloarthritis Research Consortium of Canada; UPA: upadacitinib; W: week

In patients with SPARCC index >0 at baseline, there was an improvement in patients’ assessment of pain at weeks 12, 16 and 24 in the upadacitinib 15 mg group *vs* the placebo group (nominal *P* *<* 0.0001 at each time point; Fig. 6). Improvements in pain were comparable between upadacitinib 15 mg and adalimumab 40 mg every other week (nominal *P* > 0.05 at each time point), and between the upadacitinib 15 mg and placebo to upadacitinib 15 mg groups at weeks 36 and 56. There was a relatively weak correlation between enthesitis and pain in the upadacitinib 15 mg group at each time point from baseline through week 56 [Pearson correlation coefficient at baseline: 0.259 (*n *=* *493), week 12: 0.141 (*n *=* *480), week 16: 0.226 (*n *=* *466), week 24: 0.229 (*n *=* *459), week 36: 0.196 (*n *=* *441) and week 56: 0.232 (*n *=* *418)]. However, a faster and larger improvement in pain was seen in patients with enthesitis resolution at week 24 compared with patients without resolution across all groups (Supplementary Fig. S2A and B, available at *Rheumatology* online).

## Discussion

In this *post hoc* analysis of pooled data from patients with PsA and an inadequate response or intolerance to one or more prior non-bDMARD (SELECT-PsA 1) or bDMARD (SELECT-PsA 2), upadacitinib 15 mg led to higher rates of enthesitis resolution and greater improvements in both the LEI and the more sensitive SPARCC index compared with placebo over 24 weeks of treatment, with improvements maintained through 56 weeks.

The improvements in enthesitis measures observed at week 12 in the upadacitinib 15 mg group and at week 36 in the placebo to upadacitinib 15 mg group (i.e. after 12 weeks of upadacitinib treatment) suggest a rapid onset of improvement of enthesitis with upadacitinib. Notably, the change from baseline in LEI and SPARCC index, and rates of enthesitis resolution, were similar at week 56 in patients who switched from placebo to upadacitinib 15 mg (i.e. after 32 weeks upadacitinib treatment) and those who received continuous upadacitinib from baseline. Taken together with the primary analyses, which found that upadacitinib 15 mg appeared to have achieved full effect on the resolution of enthesitis by week 16 (SELECT-PsA 1 [9]) and week 12 (SELECT-PsA 2 [10]), there is evidence that the main effect of upadacitinib treatment on resolution of enthesitis occurs within 12–16 weeks of treatment.

In this analysis, over 90% of patients receiving upadacitinib 15 mg without enthesitis at baseline were enthesitis-free at week 56, and upadacitinib 15 mg prevented recurrence of enthesitis in >80% of patients with enthesitis at baseline who achieved resolution at week 24 (LEI = 0). Analysis of residual enthesitis across the sites measured for the derivation of the LEI and SPARCC index did not reveal any apparent pattern of refractory sites, and numerically lower proportions of patients experienced enthesitis at each site with upadacitinib *vs* placebo at every LEI and SPARCC index site.

Enthesitis is a dimension of PsA that is highly relevant to patients in terms of their daily functioning, in part due to its association with pain [1, 3]. This is illustrated in our analysis by the faster and larger improvements in pain in patients who achieved enthesitis resolution at week 24 compared with those who did not achieve resolution. Improvements from baseline in pain were substantially better after 24 weeks of treatment in the upadacitinib 15 mg group (–2.78) compared with placebo (–1.19) and continued to improve up to week 56 (–3.11); a rapid improvement was also seen in the first 12 weeks after switching from placebo to upadacitinib. In our analysis, upadacitinib and adalimumab demonstrated similar improvements in pain through week 56 in patients with enthesitis; in the SELECT-PsA 1 open-label extension study [11], a trend was observed at later time points up to 104 weeks for numerically better improvements in pain and greater proportions of patients achieving resolution of enthesitis with upadacitinib 15 mg than adalimumab every other week. However, the week 104 analysis did not assess the correlation between enthesitis and pain, and further work is needed to understand the relative effects of these treatments on enthesitis and pain in PsA.

Pre-clinical studies suggest a fundamental role for JAK signalling in the pathogenesis of enthesitis [14], and upadacitinib has been shown to inhibit the phosphorylation of the signalling molecule STAT1 in entheseal cells, leading to reduced production of the inflammatory cytokines TNF and IL-17 [15]. Therefore, it is plausible that patients receiving JAK inhibitors may have improvement in enthesitis-related clinical outcomes because of reduced production of cytokines involved in development of enthesitis. In keeping with this, improvements in enthesitis have been observed with other JAK inhibitors besides upadacitinib, including a Phase 3 study of tofacitinib in patients with an inadequate response to csDMARDs [16] and a Phase 2 open-label extension study of filgotinib in patients with active PsA [17]. However, the improvements in enthesitis with tofacitinib *vs* placebo in a study of patients with inadequate response to TNF inhibitors failed to achieve statistical significance [18], potentially suggesting that different JAK inhibitors may affect enthesitis to different extents. These differences may be attributable to the variation in selectivity between upadacitinib (which is JAK1 selective) and tofacitinib (a pan-JAK inhibitor) on the regulation of cytokine signalling [19, 20]. However, the analysis reported here was *post hoc* and descriptive, and comparisons with other studies should be made with caution. Further studies that are specifically designed to evaluate and compare the effect of JAK inhibition on the development of enthesitis are needed.

Limitations of this analysis include the *post hoc* and descriptive nature of the analysis, which limits interpretation of the data to hypothesis generation only, with no investigation of the impact of other relevant factors on enthesitis beyond upadacitinib treatment, such as concomitant medications. Furthermore, no correlation analysis was conducted between enthesitis status and other measures of disease activity. The earliest data collection for enthesitis was at week 12, meaning it was not possible to assess the onset of treatment effect before this time point.

## Conclusion

This *post hoc* analysis expands on the primary analyses of the SELECT-PsA 1 and 2 studies, demonstrating that upadacitinib is associated with a comprehensive improvement in enthesitis across individual entheseal sites, with the main treatment effect observed as early as 12 weeks after initiation. Upadacitinib is associated with maintenance of an enthesitis-free state after resolution and protection against new-onset enthesitis in patients who did not have enthesitis at baseline.

## Supplementary Material

keae057_Supplementary_Data

## Data Availability

AbbVie is committed to responsible data sharing regarding the clinical trials we sponsor. This includes access to anonymized, individual and trial-level data (analysis data sets), as well as other information (e.g. protocols, clinical study reports or analysis plans), as long as the trials are not part of an ongoing or planned regulatory submission. This includes requests for clinical trial data for unlicensed products and indications. These clinical trial data can be requested by any qualified researchers who engage in rigorous, independent, scientific research, and will be provided following review and approval of a research proposal, Statistical Analysis Plan (SAP) and execution of a Data Sharing Agreement (DSA). Data requests can be submitted at any time after approval in the USA and Europe and after acceptance of this manuscript for publication. The data will be accessible for 12 months, with possible extensions considered. For more information on the process or to submit a request, visit the following link: https://www.abbvieclinicaltrials.com/hcp/data-sharing.
