# Supplementary material for: Effects of upadacitinib on enthesitis in patients with psoriatic arthritis: a post hoc analysis of SELECT-PsA 1 and 2 trials
Source: Rheumatology (Oxford). 2024 Feb 8;63(11):3146–54. doi: 10.1093/rheumatology/keae057 (PMC11534117; doi:10.1093/rheumatology/keae057)
Supplement: keae057_Supplementary_Data [file keae057_supplementary_data.zip › keae057_Supplementary_Data/rhe-23-1387-File010.docx]

# Supplementary material

**Supplementary Table S1.** Patients with and without enthesitis at baseline

| *n* (%) | Placebo *n* = 635^a^ | Placebo to  UPA 15 mg QD *n* = 317^a^ | UPA 15 mg QD *n* = 639^b^ |
| --- | --- | --- | --- |
| Enthesitis (LEI >0) | 385 (60.6) | 189 (59.6) | 403 (63.1) |
| No enthesitis (LEI=0) | 250 (39.4) | 128 (40.4) | 236 (36.9) |
| Enthesitis (SPARCC index >0) | 492 (77.5) | 244 (77.0) | 500 (78.2) |
| No enthesitis (SPARCC index =0) | 143 (22.5) | 73 (23.0) | 139 (21.8) |

^a^The placebo group includes patients who subsequently received either UPA 15 mg QD or UPA 30 mg QD from week 24; the placebo to UPA 15 mg QD group represents the subset of the placebo group who received UPA 15 mg QD from week 24 onwards. ^b^LEI or SPARCC index missing in one patient.
LEI: Leeds Enthesitis Index; QD: once daily; SPARCC: Spondyloarthritis Research Consortium of Canada; UPA: upadacitinib.

**Supplementary Table S2.** Baseline demographics and clinical characteristics in patients with and without enthesitis at baseline

| Mean (SD) unless otherwise specified | Baseline LEI >0 | | | Baseline LEI=0 | | | Baseline SPARCC index >0 | | | Baseline SPARCC index =0 | | |
| --- | --- | --- | --- | --- | --- | --- | --- | --- | --- | --- | --- | --- |
|  | **UPA  15 mg QD (*n* = 403)** | **Placebo (*n* = 385)** | **All (*n* = 788)** | **UPA  15 mg QD (*n* = 236)** | **Placebo (*n* = 250)** | **All (*n* = 486)** | **UPA  15 mg QD (*n* = 500)** | **Placebo (*n* = 492)** | **All (*n* = 992)** | **UPA 15 mg QD (*n* = 139)** | **Placebo**  **(*n* = 143)** | **All (*n* = 282)** |
| Female sex, n (%) | 241 (59.8) | 217 (56.4) | 458 (58.1) | 110 (46.6) | 114 (45.6) | 224 (46.1) | 282 (56.4) | 271 (55.1) | 553 (55.7) | 69  (49.6) | 60  (42.0) | 129 (45.7) |
| Age, years | 52.9 (12.0) | 51.7 (11.7) | 52.3 (11.9) | 50.4 (12.3) | 51.5 (12.7) | 51.0 (12.5) | 52.4 (12.0) | 51.6 (11.8) | 52.0 (11.9) | 50.7 (12.8) | 51.6 (13.2) | 51.1 (13.0) |
| BMI, kg/m^2^ | 31.1 (6.9) | 31.5 (7.2) | 31.3 (7.1) | 29.7 (6.5) | [N=249]  29.8 (6.6) | [N=485]  29.8 (6.6) | 31.0 (7.0) | 31.2 (7.2) | 31.1 (7.1) | 29.3 (5.8) | [N=142]  29.5 (6.4) | [N=281]  29.4 (6.10) |
| Duration of PsA symptoms, years | 10.2 (8.8) | [N=382] 11.4 (10.1) | [N=785] 10.8 (9.5) | 10.3 (8.9) | 10.5 (9.9) | 10.4 (9.4) | 10.4 (8.9) | [n=489] 11.2 (10.2) | [N=989] 10.8 (9.6) | 9.7  (8.3) | 10.4 (9.4) | 10.0 (8.9) |
| Duration of PsA diagnosis, years | 7.1  (7.8) | 8.0  (8.5) | 7.6  (8.2) | 7.7  (8.0) | 7.5  (8.6) | 7.6  (8.3) | 7.5  (8.2) | 7.9  (8.4) | 7.7  (8.3) | 6.9  (6.8) | 7.6  (9.0) | 7.3 (7.9) |
| TJC68 | 25.2 (16.7) | 26.6 (16.6) | 25.9 (16.7) | 16.0 (11.7) | 14.3 (10.6) | 15.2 (11.1) | 23.6 (16.2) | 24.1 (16.1) | 23.8 (16.1) | 15.4 (11.8) | 13.8 (11.2) | 14.6 (11.5) |
| SJC66 | 12.1 (9.8) | 12.5 (9.1) | 12.3 (9.5) | 10.4 (7.1) | 9.6  (7.0) | 10.0 (7.0) | 11.9 (9.4) | 11.9 (8.6) | 11.9 (9.0) | 10.1 (6.9) | 9.6  (7.6) | 9.9 (7.2) |
| Patient’s assessment of pain | [N=397]  6.5  (2.1) | [N=383]  6.5  (2.0) | [N=780]  6.5  (2.1) | [N=235]  5.8  (2.1) | [N=247]  5.9  (2.3) | [N=482]  5.9  (2.2) | [N=494]  6.3  (2.1) | [N=487]  6.5  (2.1) | [N=981]  6.4  (2.1) | [N=138]  5.9  (2.0) | 5.5  (2.3) | [N=281]  5.7 (2.2) |
| hsCRP, mg/L | 10.7 (17.0) | 11.2 (16.5) | 10.9 (16.7) | 11.7 (14.8) | 11.0 (17.1) | 11.3 (16.0) | 10.7 (16.6) | 11.4 (17.0) | 11.1 (16.8) | 12.3 (14.9) | 10.2 (15.7) | 11.2 (15.3) |
| LEI | 2.8  (1.6) | 2.9  (1.6) | 2.8  (1.6) | 0  (0) | 0  (0) | 0  (0) | 2.2  (1.8) | 2.3  (1.9) | 2.2  (1.8) | 0.1  (0.3) | 0  (0.3) | 0  (0.3) |
| SPARCC index | 6.3  (4.2) | 6.7  (4.1) | 6.5  (4.1) | 1.2  (1.7) | 1.1  (1.6) | 1.1  (1.7) | 5.7  (4.1) | 5.8  (4.0) | 5.7  (4.1) | 0  (0) | 0  (0) | 0  (0) |

BMI: body mass index; hsCRP: high-sensitivity C-reactive protein; LEI: Leeds Enthesitis Index; PsA: psoriatic arthritis; QD: once daily; SJC66: 66-swollen joint count; SD: standard deviation; SPARCC: Spondyloarthritis Research Consortium of Canada; TJC68: 68-tender joint count; UPA: upadacitinib.
