# Supplementary figures and images for: Effects of upadacitinib on enthesitis in patients with psoriatic arthritis: a post hoc analysis of SELECT-PsA 1 and 2 trials
Source: Rheumatology (Oxford). 2024 Feb 8;63(11):3146–54. doi: 10.1093/rheumatology/keae057 (PMC11534117; doi:10.1093/rheumatology/keae057)

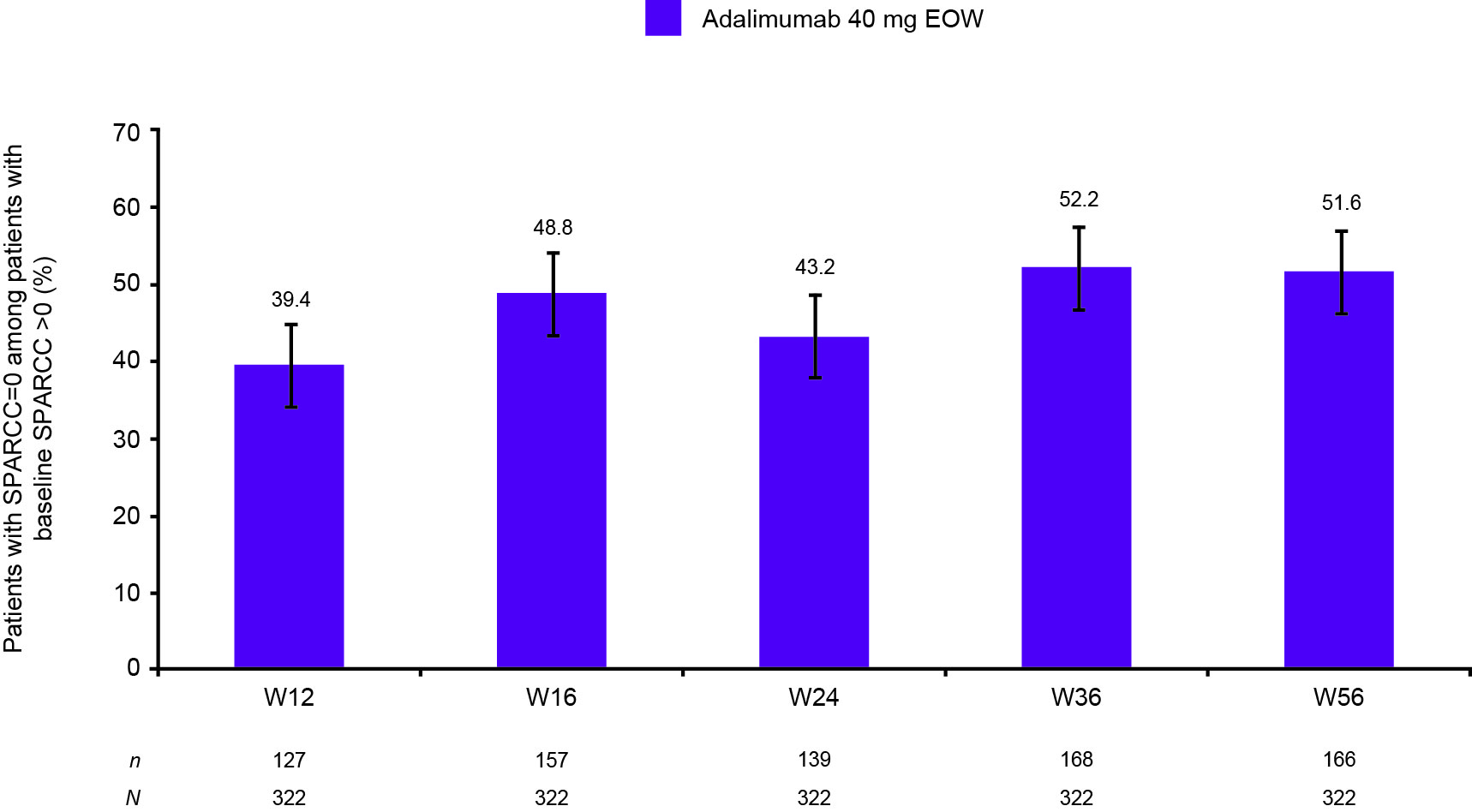

Supplement: keae057_Supplementary_Data [file keae057_supplementary_data.zip › keae057_Supplementary_Data/rhe-23-1387-File008.jpg]

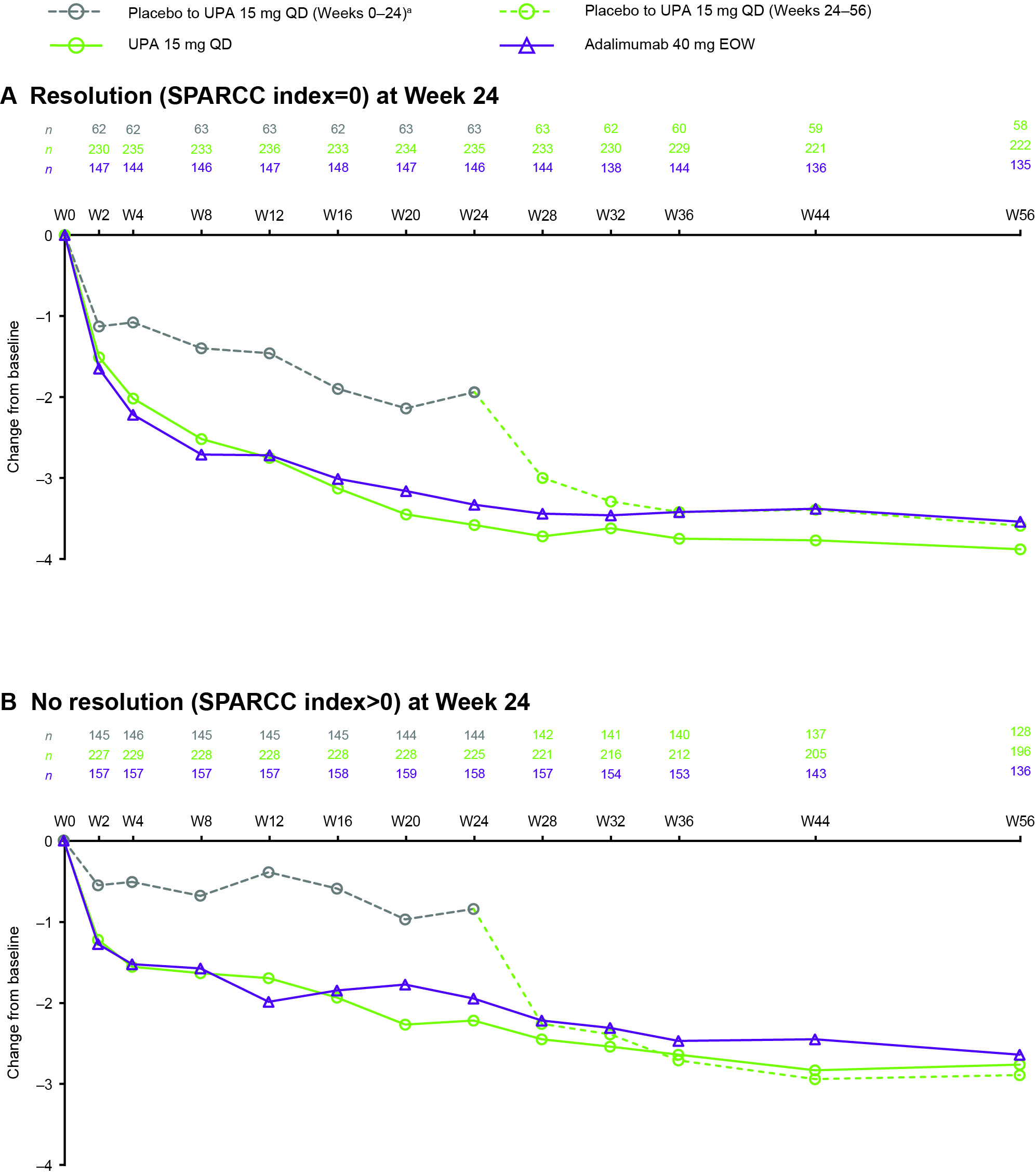

Supplement: keae057_Supplementary_Data [file keae057_supplementary_data.zip › keae057_Supplementary_Data/rhe-23-1387-File009.jpg]
